# Binge eating disorder during COVID-19

**DOI:** 10.1515/biol-2022-0033

**Published:** 2022-04-05

**Authors:** Sara Mumtaz, Syeda Mehpara Farhat, Rida Fatima Saeed, Sidra Younis, Mahwish Ali

**Affiliations:** Faculty of Multidisciplinary studies, Department of Biological Sciences, National University of Medical Sciences, Rawalpindi, Pakistan

**Keywords:** COVID, health, nutrition, syndrome

## Abstract

With the onset of coronavirus disease in December 2019, the normal routine and lifestyle of the humans has adversely affected all over the world. This change in lifestyle not only increased the level of stress and anxiety, but also badly modified the eating habits during the lockdown period. This increased the rate of binge eating disorder in people who were already immune-compromised. This rapid communication aims to develop awareness among people to stay calm during this pandemic and eat healthy.

Eating disorders are a type of mental disorder that affects physical health and psychosocial functioning. Among different eating disorders, binge eating disorder (BED) is the most prevalent malady and has been included among the seven major eating disorders (according to the international disease classification systems The Diagnostic and Statistical Manual of Mental Disorders, Fifth Edition (DM5) and International Classification of Diseases 11th Revision). In BED, there is a frequent intake of large quantities of food and no control over overeating. BED is even more common than human immunodeficiency virus (HIV), breast cancer, and schizophrenia. According to a recent study, there are 17.9 million people affected by BED in the USA [1,2]. Despite its high prevalence, BED is usually underrepresented because it is associated with considerable stigma and self-stigmatization, which obstructs help-seeking behavior, and leads to reduced visibility and poor general awareness of this disorder in society [3].

The onset of novel coronavirus disease (COVID-19) has aggravated the burden of eating disorders due to the implementation of a “stay in place” lockdown to minimize the spread of the infection. Though this lockdown had a positive effect on viral transmission, on the other hand, it had a negative impact on individuals causing high stress, anxiety, and altered eating habits, which ultimately led to eating disorders [4]. These psychological and social stressors present a greater challenge for individuals suffering from BED. A study conducted on the electronic health record of 5.2 million individuals revealed that the number of BED cases in 2017, 2018, and 2019 did not vary much, but its diagnostic incidence in 2020 increased to 15.3%, as compared to the previous years [5]. BED is of special concern because of its association with obesity. Obesity boosts the production of adipokines and other cytokines that eventually may lead to the development of other metabolic conditions such as diabetes and arterial hypertension. Obese people infected with COVID-19 are more susceptible to severe complications and death [6].

BED has such devastating effects on mental health that it may lead to an increased risk of suicide. A study conducted on the patients after the outbreak of severe acute respiratory syndrome in 2003 showed a 26.2% rise in psychological disorders [7]. Keeping in view the synergistic severities associated with BED and COVID-19 and a likelihood of onset of the next wave of COVID-19, stringent measurements should be taken for timely management of BED. This is crucial to avoid obesity-related health risks and maintain the integrity of mental health. The BED mitigation can be achieved by increasing awareness in the general public to maintain a healthy lifestyle by avoiding high glycemic index carbohydrates and increasing intake of whole grain, legumes, vegetables, and fruits. It is also recommended that healthcare professionals should question the eating habits among patients even if it is not included in the general complaints. Moreover, further research is required to implement an evidence-based approach for managing eating disorders, especially BED, during the COVID-19 pandemic. According to Katzman, the “COVID-19 is a wake-up call for making eating disorders a priority” [8].

With the onset of COVID-19 in December 2019, the normal routine and lifestyle of humans were adversely affected all over the world. This lifestyle change not only increased the level of stress and anxiety but also badly modified the eating habits during the lockdown period. This increased the ratio of BED. Therefore, we suggest that it is the foremost requirement to improve awareness among the general population for mitigation and management of this disorder. The increased awareness will help to reduce the stigmatization associated with this self-inflicted disorder and improve early engagement in treatment. Moreover, opening up new research avenues in this previously underrated field of eating disorders is also vital to provide more effective treatments with precision planning and develop new rehabilitation approaches.
